# Comprehensive Knowledge towards Cervical Cancer and Associated Factors among Women in Durame Town, Southern Ethiopia

**DOI:** 10.1155/2020/4263439

**Published:** 2020-12-29

**Authors:** Biruktawit F. Woldu, Lidiya G. Lemu, Debiso E. Mandaro

**Affiliations:** ^1^Department of Midwifery, College of Medicine and Health Sciences, Wachemo University, Hossana, Ethiopia; ^2^Department of Midwifery, College of Health Sciences, Mizan Tepi University, Mizan, Ethiopia; ^3^Department of Midwifery, College of Medicine and Health Sciences, Wachemo University, Durame, Ethiopia

## Abstract

Low awareness about cervical cancer and poor screening practice are some of the contributing factors for the high burden of cervical cancer in sub-Saharan Africa. The aim of this study was to assess comprehensive knowledge towards cervical cancer and associated factors among reproductive age women visiting Durame General Hospital. Institution-based cross-sectional study was conducted in April 2019. Systematic random sampling technique was employed to select study participants. Pretested interviewer administered questionnaire was used for data collection. Binary and multiple logistic regression analysis was done. Adjusted odds ratio with a 95% CI was used to determine the presence and strength of associations between independent and outcome variable. Variables with *p* value less than 0.05 were considered as statistically significant. Among the 237 women enrolled, more than half (55.7%) have ever heard about cervical cancer. Health professionals were major source of information. Half of respondents (51.5%) had good knowledge towards cervical cancer. Urban residence (AOR = 2.28, 95% CI (1.19-4.35)), having formal employment (AOR = 2.92, 95% CI (1.53-5.59)), and knowing someone with cervical cancer (AOR = 5.21, 95% CI (2.32-11.71)) were found to have significant association with good knowledge towards cervical cancer. The comprehensive knowledge of women towards cervical cancer was found to be insufficient. Provision of community-based health education with health professionals needs to be emphasized.

## 1. Introduction

Cervical cancer is one of the reproductive organ cancers in women which commonly arises from the lower part of the uterus. The cancer is caused by sexually transmitted human papilloma virus (HPV). HPV is the most common viral infection of the reproductive tract [1]. Cervical cancer was the fourth most common cancer in women and the ninth overall, with an estimated 569,847 new cases representing 6.6% of all female's cancers. There were an estimated 311,365 deaths from cervical cancer worldwide in 2018. Nine out of ten (90%) cervical cancer deaths occurred in low- and middle-income countries [2]. In Ethiopia, according to 2018 estimate, cervical cancer was reported to be the second leading cancer diagnosis among adult women with annual 6,294 new cases and 4,844 deaths [3].

Since invasive cervical cancers are usually preceded by a long phase of preinvasive disease, it can be prevented by early detection and treatment of precancerous lesions [1]. Despite being one of the few cancers that can be prevented by simple testing, cervical cancer is affecting women in the prime of their lives causing premature suffering and death in a critically important segment of the world's population [4].

Cervical cancer screening is an essential component of women's routine health care. It is a way to detect abnormal cervical lesions and early cervical cancer. Women with abnormal results on screening need follow-up, diagnosis, and treatment in order to prevent the development of cancer or to treat cancer at an early stage. Routine cervical cancer screening has been shown to greatly reduce the number of new cervical cancer diagnosis and deaths each year [5, 6]. World Health Organization (WHO) has recommended screening for every woman from age 30 and regularly afterwards with either of approved screening tests (HPV testing, cytology, and visual inspection with acetic acid (VIA)). The frequency of screening depends on the screening test used [6].

High prevalence of HPV infection, low awareness about cervical cancer, and poor screening practice are some of the contributing factors for the high burden of cervical cancer in sub-Saharan Africa [7]. Up to 92% of those women dying from this form of cancer have never been screened. It has also been noted that some women lack the knowledge of screening and its indications [8]. WHO global action plan for the prevention and control of noncommunicable diseases sets an objective of cervical cancer prevention through screening linked with timely treatment of precancerous lesions under the global target of 25% relative reduction in premature mortality from noncommunicable diseases by 2025 [9]. Ethiopia has set a plan to increase the proportion of women aged 30-49 years screened for cervical cancers from 0.6% to 20% [10, 11]. To achieve this plan, an assessment of comprehensive women's knowledge towards cervical cancer is considered to be important as utilization of cervical cancer screening is significantly associated with women's knowledge towards cervical cancer [12–15]. However, data on comprehensive women's knowledge towards cervical cancer is lacking in the study area. Therefore, the aim of this study was to assess the comprehensive knowledge of women towards cervical cancer and associated factors in Durame General Hospital.

## 2. Materials and Methods

Institution-based cross-sectional study was conducted in Durame General Hospital, Kembata Tambaro zone, southern Ethiopia from April 1 to 30, 2019. The hospital is located 372 km away from Addis Ababa, capital city of Ethiopia. The hospital provides family planning service for 580 women on average per month. The hospital applies a single visit approach using VIA for eligible voluntary women. All female clients aged 18 years and above visiting family planning unit in the hospital during the study period were eligible for enrollment. Sample size was calculated using single population proportion formula taking account of proportion of women having good knowledge, *P* = 53.7% [16], confidence level = 95%, margin of error = 5%, and nonresponse rate = 5%. The calculated sample size was corrected for study population, and a total of 243 women were invited to participate. Systematic random sampling technique was employed to select study participants. The first participant was selected randomly, and every 2nd women were included in the study. In case of not meeting the inclusion criteria, the next person was selected. Data were collected using pretested; the interviewer administered structured questionnaire before service provision. The data collection tool prepared in English was translated in to Amharic and then returned back to English to ensure accuracy. Before the actual data collection, pretest was done on 13 women in Nigist Elleni Mohammed Memorial Hospital, and necessary corrections made accordingly. Three trained diploma nurses collected the data supervised by one public health officer. Questions concerning risk factors, symptoms, treatment options, prevention, and early detection measures for cervical cancer were used for knowledge assessment. A score of one was given to correct answer, and a score of zero was given to the incorrect and I do not know answers. Knowledge questions were scored, and the mean score was computed. Those women scored above the mean were considered as having good knowledge and below mean as having poor knowledge [17]. Data was analyzed by using SPSS version 20. Descriptive statistics were computed. Binary logistic regression analysis was done, and all explanatory variables with *p* value less than 0.2 were entered to multiple logistic regression model. Then, the presence and strength of association was assessed using AOR and its 95% CI. And variables with *p* value < 0.05 were considered as statistically significant. Goodness of model fitness was tested by using Hosmer and Lemeshow test. Ethical approval was obtained from Wachemo University College of Medicine and Health Sciences ethical review committee with ethical approval number of *CMHS-RCS-86/2019*. Informed written consent was obtained from all respondents. Confidentiality was assured for participants.

## 3. Results

Data were analyzed from 237 women making response rate of 97.5%. Mean age of participants was 26.7 (±4.2) years. As indicated in Table 1, almost all respondents (97.9%) were married. One-fourth of women (27.4%) have attained diploma and above level of education. Around half of the participants (55.7%) were urban residents. Median parity per woman was 2 IQR[1,3]. Mean age at 1st birth was 21 (±2.37) years. Near to one-fourth of respondents (23.2%) knew a woman with cervical cancer (Table 1).

### 3.1. Knowledge towards Cervical Cancer

As displayed in Table 2, more than half (55.7%) of respondents have ever heard about cervical cancer. Major source of information were health professionals (66.4%). All women who have ever heard of cervical cancer were able to identify at least one risk factor. However, only 14.4% of them identified infection with HPV as risk factor for cervical cancer. Among those who have ever heard of cervical cancer, 116 (87.9%) and 122 (92.4%) mentioned at least one symptom and one preventive measure for cervical cancer, respectively. From 132 women who have ever heard of cervical cancer, 115 (87.1%) women knew that cervical cancer is treatable if diagnosed at early stages and mentioned atleast one treatment option. Among those who have ever heard about cervical cancer, 112 (84.8%) women knew the presence of cervical cancer screening. However, majority of them were not able to mention any of the screening techniques. Overall, half of participants (51.4%) had good knowledge towards cervical cancer and its screening (Table 2).

### 3.2. Factors Associated with Knowledge towards Cervical Cancer

In the binary logistic regression analysis, age, residence, working status, educational status, having functional TV/radio, marital status, knowing someone with cervical cancer, parity, and age at first birth were associated with good level of knowledge towards cervical cancer at *p* < 0.2 (supplementary table (available here)). In multiple logistic regression analysis, residence, working status, and knowing someone with cervical cancer were found to have significant association with good level of knowledge towards cervical cancer. Urban resident women were 2.2 times more likely to have good knowledge towards cervical cancer (AOR = 2.280, 95% CI (1.195-4.350)). Those women who have formal employment were 2.9 times more likely to have good knowledge (AOR = 2.927, 95% CI (1.532-5.594)). Those women who knew someone with cervical cancer were 5.2 times more likely to have good knowledge towards cervical cancer (AOR = 5.218, 95% CI (2.324-11.718)) (Table 3).

## 4. Discussion

This study was conducted to assess the comprehensive knowledge of women towards cervical cancer and associated factors in Durame General Hospital. In this study, more than half of participants (55.7%, 95% CI (49.4%-62%)) have ever heard about cervical cancer. This finding is comparable with a study done in Dessie [18], where 57.7% of women have heard about cervical cancer. It is higher than a study in Kenya [19], Cameroon [20], and Finote Selam [21]. This might be due to institution-based nature of this study and variation on participants' characteristics. However, it is lower than a study in Gondar [22], South Africa [23], and Qatar [24]. This indicates the need for awareness creation programs in the study area. A study in Kenya also indicated that 84% of participants have ever heard of cervical cancer which is much higher than our study. The possible explanation can be the practice of nurses and doctors towards informing every women on every visit to the hospital. Health care providers were major source of information, which is consistent with a study in Kenya [19]. This indicates that health professionals can play a significant role in educating women about cervical cancer.

Concerning knowledge towards the risk factors of cervical cancer, consistent result was reported from a study done in Dessie [18]. Having multiple sexual partners was the most identified risk factor for cervical cancer in both studies. And very few women mentioned infection with HPV as a risk factor for cervical cancer. This indicates the need for health education on HPV, the major risk factor for cervical cancer, in order to enhance the up taking of vaccines protecting against common cancer-causing types of HPV and significantly reduce the risk of cervical cancer.

Regarding awareness towards symptoms of cervical cancer, less than half of participants were able to mention at least one symptom. It is in line with another study [18]. This emphasizes the need for health education on the symptoms of cervical cancer as it helps women to recognize the disease and seek care early.

Half of the participants (52%) reported that cervical cancer could not be treated even if diagnosed at early stage which is lower than a study done in Finote Selam [21]. The possible explanation can be time variation between the studies. The increased attention given for the problem and information dissemination through medias might possibly contributed for better awareness.

In this study, less than half of participants were aware of availability of cervical cancer screening, and 27.6% of them were able to mention at least one screening method. This is lower than a study in Dessie [18]. This shows the need for intervention targeted at the promotion of screening tests and service availability in order to increase its uptake.

Overall, near to half participants (51.4%, 95% CI (45%-57.7%)) have good knowledge towards cervical cancer and its screening. It is comparable with a study in Hossana [16] and in Dessie [18]. However, it is higher than a study in Gondar [25] and in Finote Selam [21]. This can be possibly explained by variation on measurement and characteristics of participants; 45.1% were illiterate in Finote Selam. Different studies indicated that educational status has significant association on level of knowledge towards cervical cancer [18, 26–28].

In this study, urban resident women were found to have good knowledge compared with rural resident women. It is consistent with a study in Congo which indicated significant association between residence and good knowledge towards cervical cancer [26]. This can be possibly explained by the fact that urban residents have better access to health information due to better access to health institutions. Those women who have formal employment were more likely to have good knowledge towards cervical cancer. It is consistent with another study [26]. The possible justification is that working women are better educated and have better access to information. Those women who knew someone with cervical cancer were more likely to have good knowledge. It is in line with a study in Finote Selam [21]. The possible explanation is that knowing someone with cervical cancer encourages women to inquire about the disease and make them sensitive for related information.

Data presented in the present study provided an insight on the prevalence and associated factors with women's comprehensive knowledge towards cervical cancer. It also indicated a potential strategy of involving cervical cancer patients on awareness creation programs.

## 5. Limitation

As this study was conducted in urban health facility, the finding might not be representative for the rural communities. In addition, only women who sought family planning service were interviewed; thus, the findings cannot be generalizable for those who did not seek services during the study period.

## 6. Conclusion

Nearly half of the respondents have never heard about cervical cancer. Insufficient level of knowledge towards cervical cancer was observed in this study. Provisions of community-based health education on cervical cancer and its screening through health talks with health care providers and volunteer cervical cancer patients are imperative to raise women's knowledge.

## Figures and Tables

**Table 1:** Sociodemographic characteristic of female client visiting family planning unit in Durame General Hospital, 2019 (*n* = 237).

Variable	Frequency	Percentage
Age		
21-25	107	45.1
26-30	91	38.4
31-35	32	13.5
36-40	7	3.0
Marital status		
Married	232	97.9
Unmarried	5	2.1
Educational status		
No formal education	76	32.1
Primary	39	16.5
Secondary	57	24.1
Diploma and above	65	27.4
Occupation		
House wife	136	57.4
Government employee	50	21.1
Private employee	25	10.5
Merchant	26	11.0
Residence		
Urban	132	55.7
Rural	105	44.3
Have functional TV/radio		
Yes	150	63.3
No	87	36.7
Monthly income		
> 15$	53	22.4
15-30$	73	30.8
>30%$	111	46.8
Parity		
1-2	160	67.5
3-4	66	27.8
≥5	11	4.6
Age at first birth		
≥18	231	97.5
<18	6	2.5
Know someone with cervical cancer		
Yes	55	23.2
No	182	76.8

**Table 2:** Knowledge towards cervical cancer and its screening among female client visiting family planning unit in Durame General Hospital, 2019 (*n* = 132).

Variable		Frequency	Percentage
Ever heard about cervical cancer (*n* = 237)	Yes	132	55.7
	No	105	44.3

Source of information	Mass media	51	38.6
	Printed materials	5	3.8
	Health professionals	85	64.4
	Family/friends	6	4.8

Risk factors for cervical cancer	Multiple sexual partners	85	64.4
	Early sexual initiation	13	9.8
	Exposure to HPV	19	14.4
	Smoking	23	17.4
	Prolonged combined oral contraceptive use	7	5.3

Symptoms of cervical cancer	Intermenstrual bleeding	32	24.2
	Excessive vaginal discharge	14	10.6
	Foul smelling vaginal discharge	25	18.9
	Pain during/after sex	12	9.1
	Vaginal bleeding	31	23.5

Know cervical cancer can be cured if diagnosed early	Yes	114	86.4
	No	18	13.6

Treatment options	Traditional medicine	2	1.5
	Surgery	31	23.4
	Radiation	14	10.6
	Chemotherapy	74	56.1
	Do not know	15	11.4

Know availability of cervical cancer screening	Yes	112	84.8
	No	20	15.2

Screening methods (*n* = 112)	VIA	14	12.5
	Pap smear	17	15.2
	Do not know	81	72.3

**Table 3:** Bivariate and multiple logistic analysis of factors associated with level of knowledge towards cervical cancer screening among women in Durame General Hospital, 2019.

Variable	Response	Knowledge towards cervical cancer		COR (95% CI)	AOR (95% CI)
		Poor, *n* (%)	Good, *n* (%)		
Residence	Rural	48 (36.4)	84 (63.6)	1	1
	Urban	67 (63.8)	38 (36.2)	3.08 (1.81-5.25)	2.28 (1.19-4.35)^∗^

Working status	Not working	71 (65.7)	37 (34.3)	1	1
	Working	44 (34.1)	85 (65.9)	3.70 (2.16-6.35)	2.92 (1.53-5.59)^∗^

Educational status	No formal education	43 (56.6)	33 (43.4)	1	1
	Primary	24 (61.5)	15 (38.5)	0.81 (0.37-1.79)	0.55 (0.22-1.36)
	Secondary	31 (54.4)	26 (45.6)	1.09 (0.54-2.18)	0.54 (0.23-1.23)
	Diploma & above	17 (26.2)	48 (73.8)	3.67 (1.79-7.52)	1.70 (0.74-3.89)

Have functional TV/radio	Yes	55 (36.7)	95 (63.3)	3.83 (2.18-6.73)	1.86 (0.95-3.66)
	No	60 (69.0)	27 (31.0)	1	1

Know someone with cervical cancer	Yes	12 (21.8)	43 (78.2)	4.67 (2.31-9.44)	5.21 (2.32-11.71)^∗∗^
	No	103 (56.6)	79 (43.4)	1	1

## Data Availability

The datasets used and/or analyzed during the current study are available from the corresponding author on reasonable request.

## Supplementary Materials

Supplementary Materials Table: binary logistic regression analysis of factors associated with level of knowledge towards cervical cancer screening among women in Durame General Hospital, 2019.
