# Supplementary material for: Comprehensive Knowledge towards Cervical Cancer and Associated Factors among Women in Durame Town, Southern Ethiopia
Source: J Cancer Epidemiol. 2020 Dec 29;2020:4263439. doi: 10.1155/2020/4263439 (PMC7785373; doi:10.1155/2020/4263439)
Supplement: Supplementary Materials — Table: binary logistic regression analysis of factors associated with level of knowledge towards cervical cancer screening among women in Durame General Hospital, 2019. [file 4263439.f1.docx]

| **Variable** | **Response** | **Knowledge towards cervical cancer** | | **COR(95%CI)** |
| --- | --- | --- | --- | --- |
|  |  | **Poor,n(%)** | **Good,n%)** |  |
| **Age** | 21-25 | 53(49.5) | 54(50.5) | 1 |
|  | 26-30 | 44(48.4) | 47(51.6) | 1.04 (.59-1.83) |
|  | 31-35 | 15(46.9) | 17(53.1) | 1.11 (.50-2.45) |
|  | 36-40 | 3(42.9) | 4(57.1) | 1.30 (.27-6.13) |
| **Residence** | Rural | 48(36.4) | 84(63.6) | 1 |
|  | Urban | 67(63.8) | 38(36.2) | 3.08(1.81-5.25) |
| **Marital status** | Single | 2(40.0) | 3(60.0) | 1 |
|  | Married | 113(48.7) | 119(51.3) | .70 (.11-4.28) |
| **Working status** | Not working | 71(65.7) | 37(34.3) | 1 |
|  | Working | 44(34.1) | 85(65.9) | 3.70(2.16-6.35) |
| **Educational status** | No formal | 43(56.6) | 33(43.4) | 1 |
|  | Primary | 24(61.5) | 15(38.5) | 0.81 (0.37-1.79) |
|  | Secondary | 31(54.4) | 26(45.6) | 1.09 (0.54-2.18) |
|  | Diploma & above | 17(26.2) | 48(73.8) | 3.67(1.79-7.52) |
| **Have functional TV/Radio** | Yes | 55(36.7) | 95(63.3) | 3.83(2.18-6.73) |
|  | No | 60(69.0) | 27(31.0) | 1 |
| **Know someone with cervical cancer** | Yes | 12(21.8) | 43(78.2) | 4.67(2.31-9.44) |
|  | No | 103(56.6) | 79(43.4) | 1 |
| **Parity** | 1-2 | 76(48.1) | 82(51.9) | 1 |
|  | 3-4 | 31(47.0) | 35(53.0) | 1.04 (.58-1.86) |
|  | >=5 | 8(61.5) | 5(38.5) | .57(.182-.84) |
| **Age at 1st birth** | <18 | 2(33.3) | 4(66.7) | 1 |
|  | >=18 | 113(48.9) | 118(51.1) | .52(.09-2.90) |

**Table:** Binary logistic regression analysis of factors associated with level of knowledge towards cervical cancer screening among women in Durame General Hospital, 2019
